# Prevalence and Risk Factors of Voice Disorders Among Teachers in Saudi Arabia

**DOI:** 10.7759/cureus.56540

**Published:** 2024-03-20

**Authors:** Nader S Alharbi, Salman Alotaibi, Azzam I Alnughaythir, Faisal Abohelaibah, Abdullah Q Alruways, Rawan Alharbi, Saud A Alzahrani, Hatim Alsaedi, Bader Alotaibi

**Affiliations:** 1 Department of Otolaryngology, Head and Neck Surgery, Shaqra University, Riyadh, SAU; 2 Department of Medicine and Surgery, Shaqra University, Riyadh, SAU; 3 Otolaryngology, Security Forces Hospital, Riyadh, SAU; 4 College of Medicine, Shaqra University, Riyadh, SAU; 5 College of Medicine, King Abdulaziz University, Jeddah, SAU; 6 Department of Otorhinolaryngology, Taibah University, Medina, SAU

**Keywords:** saudi arabia, vocal health, voice handicap index, occupational health, teachers, voice disorders

## Abstract

Introduction

Voice is a crucial tool for communication, and voice problems are more likely to occur in professionals who frequently use their voice for work. Teachers, whose profession requires sustained vocal use, are particularly susceptible to occupation-related voice disorders. This study aimed to quantify the prevalence of voice disorders among teachers in Saudi Arabia, with the general population serving as a control group, and to identify associated risk factors.

Methods

A cross-sectional study was conducted utilizing an online self-administered questionnaire, which was completed by both teachers and the general population in Saudi Arabia. The latter group acted as a control. The questionnaire included sections on sociodemographic data, teaching patterns, symptoms of voice issues, and the Voice Handicap Index-10 (VHI-10) for assessing voice disorders among participants.

Results

The study included 640 participants, with 438 (68.4%) being teachers, the majority of whom were females (N = 406; 63.4%). The most common voice-related symptoms reported by teachers were hoarseness (N = 210; 37.9%) and dry throat (N = 147; 26.9%). Voice disorders, as determined by the VHI, affected 355 (55.5%) of the teachers. A high VHI score was associated with a diagnosis of voice disorders and GERD. There was no significant difference in the VHI scores between teachers and the general population (p > 0.05).

Conclusion

Teachers in Saudi Arabia exhibited a higher prevalence of voice disorders compared to the general population. Risk factors, such as smoking, longer teaching experience, and more teaching hours per week, were more common among teachers with voice disorders. Further investigative studies are warranted to elucidate the causal relationships between these variables and voice disorders.

## Introduction

As an essential communication tool, voice is particularly critical for professionals requiring extensive vocal use [1]. Among these professionals, teachers are at a heightened risk for voice disorders due to the vocal demands of their profession [2-6]. Studies have shown that while voice problems may affect 6-15% of the general population, this prevalence increases to 20-50% and may reach as high as 80% among teachers [7-10]. The impact of voice disorders on teachers is significant, impairing their ability to communicate effectively with students and perform efficiently, which can negatively influence students' academic success [3,5,11,12].

Various risk factors contribute to the development of voice disorders in teachers, such as extended teaching hours, years of experience, stressful environmental conditions, and a family history of vocal issues. In addition, personal attributes, such as age, gender, allergies, and smoking habits, along with speaking loudly, have been identified as contributing factors [3,11,13,14]. Although voice disorders are not life-threatening, they can adversely affect an individual's quality of life [15]. Early identification and management of voice disorders are vital to maintaining vocal health and preventing future complications [3,13].

In both research and clinical practice, self-assessment tools, such as the Voice Handicap Index (VHI), are widely used to evaluate the impact of voice disorders on the quality of life. The VHI includes 30 questions that assess the functional, physical, and emotional burdens of a voice disorder on a patient's life, with higher scores indicating greater disability [16]. The VHI-10, a shortened version of the VHI with 10 critical items, has been validated and found to correlate strongly with the full version [17]. The Arabic versions of the VHI and VHI-10 have also been validated, confirming their reliability in Arabic-speaking populations [18].

This study aims to estimate the prevalence of voice disorders among teachers in Saudi Arabia and to identify potential risk factors that may contribute to the development of such conditions. By examining these parameters, the study seeks to provide insights that could lead to better prevention strategies and targeted interventions to preserve the vocal health of teachers.

## Materials and methods

This cross-sectional study was initiated after obtaining ethical approval from Shaqra University's Institutional Review Board in June 2023 (approval number ERC_SU_20230035). The study aims to determine the prevalence of voice disorders among Saudi teachers and to determine any potential risk factors.

To calculate the appropriate number of samples for our study, we contacted the Ministry of Education and obtained the latest data. The latest information available from the Ministry's 2018 Census results estimated that Saudi Arabia has approximately 430,000 teachers. Using this population size, we determined that a sample of 384 teachers would be required for our study. This estimate is based on 95% confidence and a ±5% margin of error.

Teachers and the general population aged 22 and up were enrolled in the study after providing informed consent. Individuals under the age of 22 were excluded, as were any vocational voice users, such as lawyers, singers, actresses, and call center representatives, as well as those who had had neck, thyroid, or upper aerodigestive tract surgery.

A self-administered online survey was distributed to schools and education-related social media groups.

The Vocal Handicap Index (VHI), created by Jacobson et al. (1997), is a useful tool for assessing vocal problems [16]. It analyzes the functional, physical, and emotional effects of these illnesses using a series of 30 questions, each reflecting how severe the patient's voice-related difficulties are. The VHI scores range from 0 (never affected) to 4 (always affected), with higher scores indicating more severe voice impairment. Rosen et al. later validated the Voice Handicap Index-10 (VHI-10), a more concise version of the VHI-30. This 10-item form has been proven to be highly correlated with its original, longer version (see Appendix A) [17]. We used a validated Arabic version of the VHI, adapted by Saleem et al., to ensure that the assessment was culturally suitable for the participants (see Appendix B) [18]. In our research, a voice disorder was identified at a VHI cut-off of 13.5, resulting in a sensitivity of 0.994 and specificity of 0.989 [19].

The survey is divided into four sections, beginning with general background information and progressing to particular professional specifics. It begins with a sociodemographic portion that contains the participants' age groups, marital status, education level, smoking history, and any pertinent surgical history, such as vocal cord surgery, thyroidectomy, or parathyroidectomy (see Appendix C). The second section includes detailed questions about teaching details, such as years of experience, grade levels taught, student gender composition, whether they teach in public or private institutions, subjects they specialize in, and weekly teaching load (see Appendix D). The third section focuses on identifying any symptoms of voice problems that the respondents might have experienced. The fourth section employs the Voice Handicap Index (VHI) to assess the severity of voice disorders, providing a quantitative measure of the impact on the participants' vocal health.

Version 26 of the Statistical Packages for the Social Sciences (SPSS) software was used to analyze the data. For categorical variables, descriptive statistics were reported as frequencies and percentages, whereas means and standard deviations were used for continuous variables. The Mann-Whitney U and Kruskal-Wallis H tests were used to compare VHI scores and domains based on socio-demographic factors and instructional experiences. The normality of distribution was examined using the Shapiro-Wilk and Kolmogorov-Smirnov tests, which revealed a non-normal distribution for VHI scores; hence, non-parametric tests were used. Statistical significance was defined as a p-value less than 0.05.

## Results

In total, 640 participants were enrolled (438 teachers vs. 202 general population). Table 1 presents the sociodemographic characteristics of participants. Overall, 233 (36.4%) were aged between 40 and 49 years old, with females being dominant. Respondents who were living in the Western Region constituted 269 (42%). For marital status, 453 (70.8%) were married. In terms of education, 449 (70.2%) were bachelor's degree holders. Only 82 (12.8%) were smokers. The perceived voice disorder has been reported by 128 (20%) of participants (N = 107, 24.4% teachers vs. N = 21, 10.4% general population), while those who were diagnosed with gastroesophageal reflux disease (GERD) were 143 (22.3%). Further details of the sociodemographic characteristics between teachers and the general population are discussed in detail in Table 1.

**Table 1:** Sociodemographic characteristics between the teachers and the general population Results are expressed as numbers and percentages (%).

Study data	Overall N (%) (^n=640)^		
		Teachers N (%) ^(n=438)^	General population N (%) ^(n=202)^
Age group			
22–29 years	177 (27.7%)	66 (15.1%)	111 (55.0%)
30–39 years	148 (23.1%)	107 (24.4%)	41 (20.3%)
40–49 years	233 (36.4%)	196 (44.7%)	37 (18.3%)
≥50 years	82 (12.8%)	69 (15.8%)	13 (06.4%)
Gender			
Male	234 (36.6%)	160 (36.5%)	74 (36.6%)
Female	406 (63.4%)	278 (63.5%)	128 (63.4%)
Residence region			
Central Region	109 (17.0%)	63 (14.4%)	46 (22.8%)
Eastern Region	76 (11.9%)	64 (14.6%)	12 (05.9%)
Western Region	269 (42.0%)	169 (38.6%)	100 (49.5%)
Southern Region	55 (08.6%)	51 (11.6%)	04 (02.0%)
Northern Region	131 (20.5%)	91 (20.8%)	40 (19.8%)
Marital status			
Single	187 (29.2%)	83 (18.9%)	104 (51.5%)
Married	453 (70.8%)	355 (81.1%)	98 (48.5%)
Educational level			
Primary school	04 (0.60%)	01 (02.0%)	03 (01.5%)
Middle school	05 (0.80%)	03 (0.70%)	02 (01.0%)
High school	38 (05.9%)	24 (05.5%)	14 (06.9%)
Diploma	61 (09.5%)	46 (10.5%)	15 (07.4%)
Bachelor's degree	449 (70.2%)	310 (70.8%)	139 (68.8%)
Masteral degree	45 (07.0%)	32 (07.3%)	13 (06.4%)
PhD	38 (05.9%)	22 (05.0%)	16 (07.9%)
Smoking			
Yes	82 (12.8%)	47 (10.7%)	35 (17.3%)
No	558 (87.2%)	391 (89.3%)	167 (82.7%)
Do you have a voice disorder?			
Yes	128 (20.0%)	107 (24.4%)	21 (10.4%)
No	512 (80.0%)	331 (75.6%)	181 (89.6%)
Do you have GERD?			
Yes	143 (22.3%)	101 (23.1%)	42 (20.8%)
No	497 (77.7%)	337 [76.9%]	160 [79.2%]

The occupational information of the teachers, as presented in Table 2, indicates that 157 (35.8%) have 11-20 years of teaching experience. A significant portion (179, 40.9%) teaches at the primary school level, and nearly half of these educators (N = 215, 49.1%) predominantly teach female students. Notably, 381 (87%) are employed by government schools, and 198 (45.2%) of the sample teach in classrooms with 21-30 students. Mathematics is the most commonly taught subject by these teachers, representing 74 (16.9%) of the cohort. In addition, 265 (60.5%) of the teachers handle 10-20 classes weekly, with the majority (N = 346, 79%) having each class last 45 minutes or less, and 256 (58.4%) have experience teaching at different educational levels.

**Table 2:** Teaching characteristics of the teachers Results are expressed as numbers and percentages (%).

Variables	N (%)
Years of experience in teaching	
<5 years	78 (17.8%)
5–10 years	65 (14.8%)
11–20 years	157 (35.8%)
>20 years	138 (31.5%)
Teaching educational level	
Primary school	179 (40.9%)
Middle school	96 (21.9%)
High school	111 (25.3%)
University	52 (11.9%)
Gender taught	
Male	147 (33.6%)
Female	215 (49.1%)
Both	76 (17.4%)
Type of school	
Government (Public)	381 (87.0%)
Private	57 (13.0%)
Number of students per class	
<20 students	45 (10.3%)
21–30 students	198 (45.2%)
>30 students	195 (44.5%)
Subject taught	
Science	63 (14.4%)
English language	47 (10.7%)
Arabic language	66 (15.1%)
Social science	66 (15.1%)
Mathematics	74 (16.9%)
Islamic studies	62 (14.2%)
Art	02 (0.50%)
Physical education	14 (03.2%)
Computer science	26 (05.9%)
All subjects	18 (04.1%)
Number of classes taught per week	
<10 class	85 (19.4%)
10–20 class	265 (60.5%)
>10 class	88 (20.1%)
The estimated time per class in minutes	
≤45 minutes	346 (79.0%)
>45 minutes	92 (21.0%)
Any teaching experience at different levels before	
Yes	256 (58.4%)
No	182 (41.6%)

In examining the VHI, an average score of 20.3 with a standard deviation of 13.3 was noted. The mean scores across the functional, physical, and emotional domains were 7.73, 6.27, and 6.29, respectively. A comparison of VHI scores between teachers and the general population showed no statistically significant differences across the functional, physical, and emotional domains or the total VHI score. For further details, refer to Table 3.

**Table 3:** Descriptive statistics of the Voice Handicap Index between the teachers and the general population VHI, Voice Handicap Index Results are expressed as mean ± SD. § P-value has been calculated using Mann-Whitney Z-test.

VHI domain	Overall Mean ± SD ^(n=640)^	Teacher		P-value ^§^
		Yes Mean ± SD ^ [n=438]^	No Mean ± SD ^ [n=202]^	
Functional score	7.73 ± 5.37	7.89 ± 5.47	7.37 ± 5.12	0.191
Physical score	6.27 ± 4.54	6.55 ± 4.79	5.66 ± 3.87	0.066
Emotional score	6.29 ± 4.08	6.47 ± 4.29	5.89 ± 3.58	1.501
Total VHI score	20.3 ± 13.3	20.9 ± 13.8	18.9 ± 11.8	0.078

**Figure 1:**
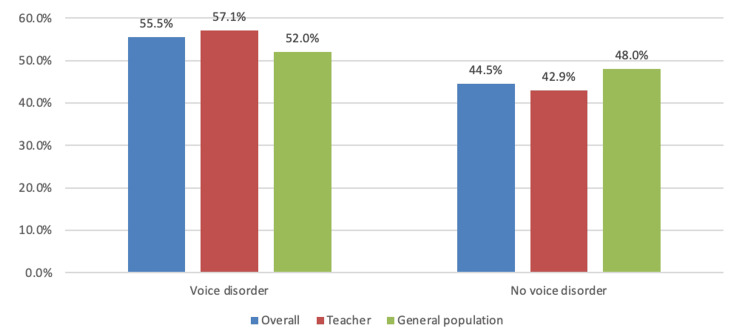
Prevalence of voice disorders between the teachers and the general population Data are expressed as numbers and percentages (%).

The prevalence of voice disorders, as illustrated in Figure 1, indicates that 355 (55.5%) of the participants were affected based on the VHI criteria. Comparing teachers with the general population revealed a higher incidence of voice disorders among teachers, at 250 (57.1%), compared to 105 (52%) in the general population.

**Figure 2:**
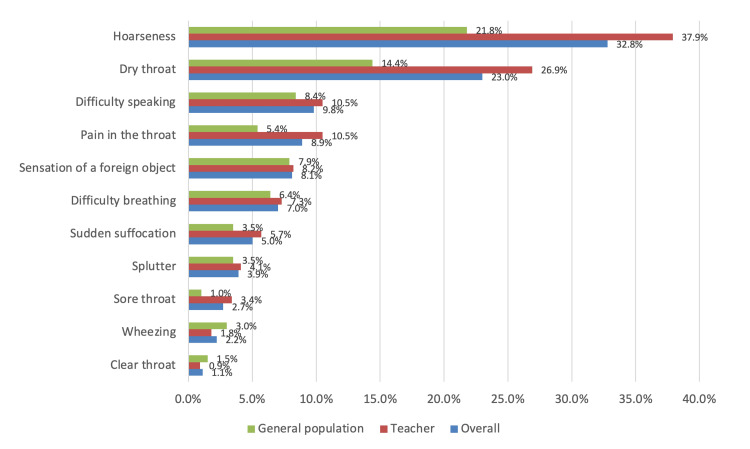
Symptoms associated with voice problems Data are expressed as numbers and percentages (%).

Figure 2 provides an overview of the symptoms associated with voice problems. The most frequently reported symptom among respondents was hoarseness at 210 (32.8%), followed by a dry throat at 147 (23%) and difficulty speaking at 63 (9.8%). Specifically, among teachers, hoarseness was reported at a higher rate of 166 (37.9%), dry throat at 118 (26.9%), and throat pain at 46 (10.5%). By contrast, the general population reported hoarseness (N = 44, 21.8%), dry throat (N = 29, 14.4%), and difficulty speaking (N = 17, 8.4%) as the most common symptoms.

When measuring the association between VHI scores and the teachers' sociodemographic characteristics (Table 4), a higher VHI score was more associated with being a smoker (Z = 2.524, p = 0.012), having a voice disorder (Z = 9.004, p < 0.001), and being diagnosed with GERD (Z = 5.355, p < 0.001). Compared to the general population, a higher VHI score was more associated with having a voice disorder (Z = 5.334, p < 0.001) and being diagnosed with GERD (Z = 2.235, p = 0.025).

**Table 4:** Association between the overall VHI score and sociodemographic characteristics of the participants (n = 640) Results are expressed as mean ± SD. a P-value has been calculated using Mann-Whitney Z-test. b P-value has been calculated using Kruskal-Wallis H-test. * Significant at the p < 0.05 level.

Factor	Teacher ^ (n=438)^			General population ^ (n=202)^		
	VHI score (80) Mean ± SD	Z/H-test	P-value	VHI score (80) Mean ± SD	Z/H-test	P-value
Age group ^a^						
<40 years	21.0 ± 13.9	0.062	0.950	18.8 ± 11.1	0.535	0.592
≥40 years	20.8 ± 13.8			19.4 ± 13.9		
Gender ^a^						
Male	22.6 ± 15.4	1.446	0.148	19.8 ± 13.8	0.227	0.820
Female	19.9 ± 12.8			18.4 ± 10.5		
Residence region ^a^						
Inside Western Region	21.2 ± 14.2	0.375	0.708	17.9 ± 9.52	0.224	0.822
Outside Western Region	20.2 ± 12.9			21.3 ± 16.0		
Marital status ^a^						
Single	21.6 ± 15.5	0.142	0.887	19.9 ± 12.0	1.515	0.130
Married	20.8 ± 13.4			17.8 ± 11.6		
Educational level ^b^						
Diploma or below	20.9 ± 13.2	1.308	0.520	19.4 ± 11.3	0.280	0.869
Bachelor's degree	21.3 ± 14.4			18.8 ± 11.4		
Master or PhD	18.6 ± 11.4			19.1 ± 14.5		
Smoking ^a^						
Yes	25.0 ± 15.1	2.524	0.012 **	17.4 ± 9.95	0.573	0.566
No	20.4 ± 13.6			19.2 ± 12.2		
History of voice disorder ^a^						
Yes	30.9 ± 16.4	9.004	<0.001 *	32.3 ± 14.1	5.334	<0.001 *
No	17.7 ± 11.1			17.4 ± 10.5		
History of gastroesophageal reflux disease? ^a^						
Yes	27.8 ± 17.0	5.355	<0.001 *	21.8 ± 13.4	2.235;	0.025 *
No	18.8 ± 11.9			18.2 ± 11.3		

An analysis presented in Table 5 indicates a correlation between higher VHI scores and teachers with 5-10 years of experience (H = 8.247, p = 0.041), as well as those teaching a greater number of classes per week (H = 8.324, p = 0.016). However, no significant differences were found in VHI scores in relation to years of experience, educational level taught, gender composition of students, type of school, class size, subject taught, class duration, or experience teaching at various educational levels.

**Table 5:** Association between the VHI score and teaching patterns of the teachers (n = 438) Results are expressed as mean ± SD. a P-value has been calculated using Mann-Whitney Z-test. b P-value has been calculated using Kruskal-Wallis H-test.* Significant at the p < 0.05 level.

Factor	VHI score (80) Mean ± SD	Z/H-Score	P-value ^§^
Years of experience in teaching ^b^			
<5 years	19.1 ± 12.6	H=8.247	0.041 *
5–10 years	24.2 ± 15.7		
11–20 years	19.8 ± 12.6		
>20 years	21.7 ± 14.7		
Educational level taught ^b^			
Primary school	19.9 ± 12.6	H=2.538	0.468
Middle school	22.8 ± 14.9		
High school	20.8 ± 14.6		
University	21.1 ± 14.1		
Gender taught ^b^			
Male	22.6 ± 15.4	H=3.916	0.141
Female	19.6 ± 12.9		
Both	21.3 ± 13.1		
Type of school taught ^a^			
Government	20.8 ± 14.1	Z=0.948	0.343
Public	21.3 ± 12.2		
Number of students per class ^b^			
<20 students	18.6 ± 13.4	H=5.054	0.080
21–30 students	20.2 ± 13.3		
>30 students	22.2 ± 14.4		
Subject taught ^b^			
Science	23.2 ± 13.5	H=8.297	0.505
English language	24.2 ± 17.5		
Arabic language	19.5 ± 12.2		
Social science	21.2 ± 15.8		
Mathematics	20.9 ± 14.1		
Islamic studies	19.4 ± 10.6		
Art	24.0 ± 15.6		
Physical education	22.1 ± 18.5		
Computer science	18.4 ± 10.2		
All subjects	16.0 ± 9.89		
Number of classes per week ^b^			
<10 class	19.8 ± 12.3	H=8.324	0.016 *
10–20 class	20.1 ± 13.5		
>10 class	24.3 ± 15.8		
Estimated time per class in minutes ^a^			
≤45 minutes	21.4 ± 14.2	Z=1.240	0.215
>45 minutes	19.2 ± 12.0		
Any teaching experience at different levels before ^a^			
Yes	22.2 ± 15.4	Z=1.503	0.133
No	19.1 ± 11.1		

## Discussion

Researchers conducted this study to determine the prevalence of voice disorders among teachers in Saudi Arabia and identify any associated risk factors. The findings of this study revealed that based on the VHI questionnaire, the prevalence of voice disorder among teachers was 250 (57.1%), slightly higher than the general population (N = 105, 52%). A study conducted in China supported this finding [12]. Using a similar questionnaire, the prevalence of voice disorders among Chinese teachers was 47.9%. Another study in Spain confirmed this, detecting a voice disorder prevalence of 59% among kindergarten and elementary education teachers [20]. However, in Iran, the prevalence of voice problems per VHI criteria was 27.2%, which was lower than the previous reports [21]. 

Incidentally, Trinite et al. documented that two-thirds of the teachers had perceived voice problems, which was higher in females (68.2%) than males (48.8%). In our study, the difference in voice problems between male and female teachers was not significantly different (p = 0.148) [6]. However, 20% of our respondents (N = 128) believed that they had voice problems, notably higher among teachers (N = 107, 24.4%) than the general population (N = 21, 10.4%). Similarly, Seifpanahi et al. (2016) showed that more than half of teachers complained of vocal problems at work compared to nonteachers [22]. By contrast, a study done by Devadas et al. (2017) reported lower rates of self-reported voice problems at 17.4% [23].

Various risk factors contribute to voice problems. Byeon et al. (2019) identified gender, caffeine consumption, upper airway problems, speaking loudly, and number of classes per week as the most common risk factors for voice disorders and a contributing factor to resignation [3]. These corroborated the reports of de Sousa et al. (2019), who found that an increasing number of students per class was associated with an increasing risk for voice disorder [4]. Our results also reflected this, as we observed that an increase in the number of classes taught per week posed a risk factor for developing voice disorders. Furthermore, increasing years of teaching experience revealed a significant association with voice problems, which coincided with the study of Roy et al. (2004) [10].

Moreover, we have learned that smoking is another factor that increases the risk of developing voice problems. Alrahim et al. [11] and Malki et al. (2010) [13] have both observed this phenomenon. Risk factors for voice problems include smoking, acid reflux, a family history of hoarseness, and work-related stress. Byeon et al. (2019) found no significant difference between smoking, drinking alcohol, and water intake in relation to voice disorder [3].

Previous diagnoses of GERD increase the risk of voice disorders, with significant differences in both teachers and the general population (p < 0.05). Notwithstanding these results, Alva et al. (2017) found an association between voice disorders in terms of upper respiratory infections, deviated nasal septum, and GERD (p < 0.05) [14]. A study conducted in Latvia identified an increased risk of voice disorders associated with throat clearing, neglecting personal health, chronic illnesses of the upper respiratory tract, allergies, and regular workplace stress [6].

Assessing the scores of VHI and its domains, our results indicate that the functional domain has the highest mean score (7.73) compared to the emotional (6.29) or physical (6.27) domains. The mean VHI score was 20.3 out of 80 points. When we compared these results between teachers and the general population, we found no significant differences between the study population in relation to VHI and its domains (p > 0.05). Contradicting these reports, Ghayoumi-Anaraki et al. (2020) found significant differences between VHI scores and its domains according to teachers with or without voice disorders (p < 0.05) [21]. 

In a study conducted in China, hoarseness was the most commonly reported complaint related to voice problems [12]. However, chronic laryngitis, vocal cord polyps, and vocal cord nodules were the most commonly diagnosed voice disorders among Chinese teachers. In Spain, at the end of working duty, over 60% of the teachers complained of frequent vocal fatigue and 55% reported hoarseness, while in India, sore or dry throat, tired voice, neck tension, muscle pain, strain in voice, and difficulty in projecting voice were the most prominent symptoms of voice problems [20,23]. In our study, the teachers' most common symptoms were hoarseness, followed by dry throat and pain in the throat, which coincided with the symptoms of the general population, such as hoarseness and dry throat. 

Certain limitations have shaped the results of this study. The use of an online questionnaire may have introduced response biases, affecting the accuracy of the results due to the possibility of untruthful or misunderstood responses. The study's cross-sectional design only uncovers correlations, not causations, which limits our understanding of the direct impact of identified risk factors on voice disorders. Furthermore, the subjective nature of the self-reported VHI questionnaire may not accurately reflect the actual clinical severity of voice disorders.

## Conclusions

There was a high prevalence of voice disorders among teachers in Saudi Arabia. Teachers who were smokers, with increasing years of teaching and number of classes per week, were more likely to complain of voice problems than any of our population. Interestingly, both teachers and nonteachers who had perceived voice problems and GERD diagnosis were more likely to develop voice disorders. It is necessary to identify the risk factors that may influence voice disorders. The findings of this study indicate that education, treatment, and prevention programs are vital to decreasing the prevalence of voice problems related to teaching.

## Figures and Tables

**Figure 3:**
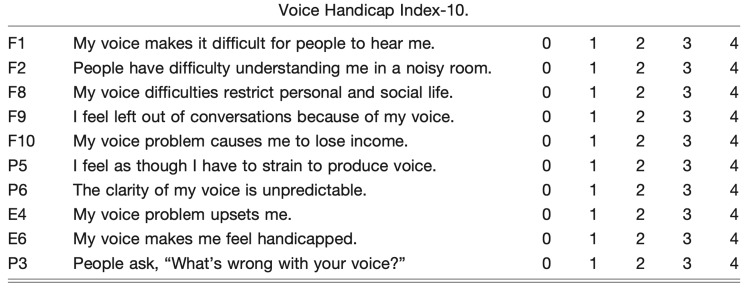
Original Voice Handicap Index-10 (VHI-10) Reference: [17]

**Figure 4:**
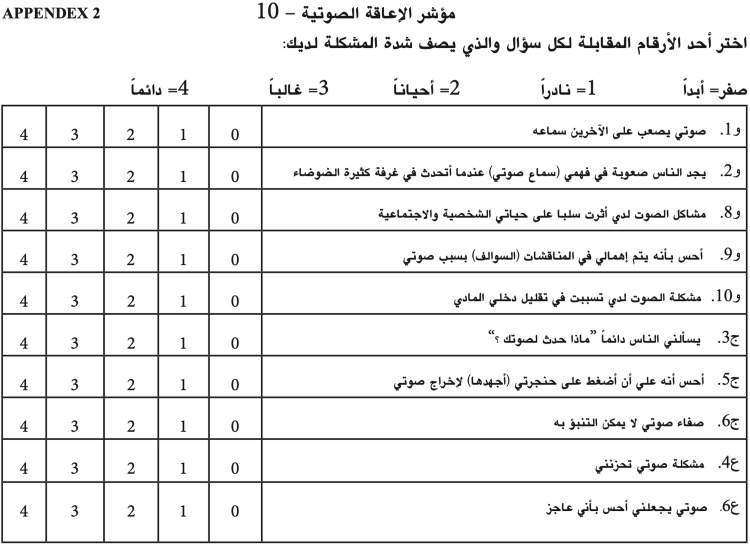
Validated Arabic version of the Voice Handicap Index-10 (VHI-10) Reference: [18]

## Appendices

Appendix A 

Appendix B 

Appendix C

**Table 6:** Sociodemographic questions

Age: 22 – 29 years 30 – 39 years 40 – 49 years ≥50 years
Gender: Male Female
Residence region: Central Region Eastern Region Western Region Southern Region Northern Region
Marital status: Single Married
Educational level: Primary School Middle School High School Diploma Bachelor’s degree Master's degree PhD
Do you smoke? Yes No

Do you have a voice disorder? Yes No
Do you have GERD (Gastroesophageal Reflux Disease)? Yes No
Have you had any surgeries in the neck or the upper aerodigestive tract before? Yes No
Do you work in a job that requires frequent use of your voice, such as: Lawyer Singer Customer service Actor Other (specify) I don’t use my voice frequently in my job.
Do you currently work as a teacher? Yes No

Appendix D

**Table 7:** Teaching characteristics questions

How many years of teaching experience do you have? <5 years 5-10 years 11-20 years >20 years

What educational level do you teach? Primary School Middle School High School University
What gender do you teach? Male Female Both
What type of school do you teach at? Government (Public) Private
How many students are there per class? <20 students 21-30 students >30 students
What subject do you teach? Science English language Arabic language Social science Mathematics Islamic studies Art Physical education Computer science All subjects
How many classes do you teach per week? <10 classes 10-20 classes >20 classes
What is the typical duration of one class (in minutes)? ≤45 minutes >45 minutes
Have you taught at any other educational level before? Yes No
